# Detection and Mitigation of GNSS Gross Errors Utilizing the CEEMD and IQR Methods to Determine Sea Surface Height Using GNSS Buoys

**DOI:** 10.3390/s25092863

**Published:** 2025-04-30

**Authors:** Jin Wang, Shiwei Yan, Rui Tu, Pengfei Zhang

**Affiliations:** 1 College of Geodesy and Geomatics, Shandong University of Science and Technology, Qingdao 266590, China; wangjin@sdust.edu.cn (J.W.); 202283020009@sdust.edu.cn (S.Y.); turui@sdust.edu.cn (R.T.); 2 Chinese Academy of Surveying and Mapping, Beijing 100830, China

**Keywords:** GNSS buoy, raw sequential coordinate series, gross error detection, CEEMD-IQR, sea surface altimetry

## Abstract

Determining the sea surface height using Global Navigation Satellite System (GNSS) buoys is an important method for satellite altimetry calibration. The buoys observe the absolute height of the sea surface using GNSS positioning technology, which is then used to correct the systematic deviation of the altimeter of the orbiting satellite. Due to the challenging observational conditions, such as significant multipath errors in GNSS code observation and complex variations in buoy position and attitude, gross errors in GNSS buoy positioning reduce the accuracy and stability of the calculated sea surface heights. To accurately detect and remove these gross errors from GNSS coordinate time series, the complementary ensemble empirical mode decomposition (CEEMD) method and the interquartile range (IQR) method were adopted to enhance the accuracy and stability of GNSS sea surface altimetry. Firstly, the raw GNSS sequential coordinate series are decomposed into main terms, such as trend contents and periodic contents, and high-frequency noise terms using the CEEMD method. Subsequently, the high-frequency noise terms of the GNSS coordinate series are regarded as the residual sequences, which are used to detect gross errors using the IQR method. This approach, which integrates the CEEMD and IQR methods, was named CEEMD-IQR and enhances the ability of the traditional IQR method to detect subtle gross errors in GNSS coordinate time series. The results indicated that the CEEMD-IQR method effectively detected gross errors in offshore GNSS coordinate time series using GNSS buoys, presenting a significant enhancement in the gross error detection rate of at least 35.3% and providing a “clean” time series for sea level measurements. The resulting GNSS sea surface altimetry accuracy was found to be better than 1.51 cm.

## 1. Introduction

Studying the ocean is crucial for advancing human marine exploration activities and economic growth as it can provide essential technical support and insights [1]. The acquisition of tidal observation data is essential for marine scientific research, ocean resource exploration and development, marine surveying, and marine disaster prevention and control [2,3,4,5]. Traditional tidal measurements typically involve the deployment of tide gauge stations, utilizing tools such as tide staffs, pressure tide gauges, acoustic water level gauges, or radar water level gauges. To obtain tidal measurement data, a leveling surveying process is required at these stations using the aforementioned methods, necessitating substantial human and material resources [6].

With the advent of various new sensors, including satellite altimetry [7], acoustic Doppler measurement technology [8], image processing technology [9], laser ranging technology [10], and radar technology [11], tidal measurement methods have diversified significantly. Among these new sensors, GNSS has been widely used for marine tidal measurements due to the advantage of it being able to observe in all weather and time conditions. It is particularly utilized in nearshore and offshore tide measurements and sea level dynamic monitoring [12,13]. GNSS tidal measurement technology can be broadly categorized into three types: GNSS-R technology, which utilizes GNSS reflected signals [14,15]; GNSS-MR technology, which employs GNSS signal-to-noise ratio observation data; and GNSS positioning technology [16,17]. For tidal measurements using GNSS-R and GNSS-MR technologies, the GNSS receiver must be stationary [14], making these techniques unsuitable for use on GNSS buoys.

Employing GNSS buoys with GNSS positioning technology has gradually become a significant method for measuring sea surface tides and monitoring waves [18]. Utilizing GNSS differential positioning technology, centimeter-level accuracy in sea surface altimetry can be achieved. For instance, the GPS system has demonstrated sub-centimeter accuracy in real-time river height monitoring [19]. GPS positioning technology, combined with the Earth gravity field model EGM08, has been used to calculate tidal changes in Greece [20]. The global rise in sea level from 1980 to 2009 was monitored using GPS positioning technology, which was consistent with the trends observed at tide gauge stations [21]. Furthermore, GPS precise positioning technology has been used to calculate tidal changes in Turkey from the late 20th to early 21st centuries, aligning with data from sea level monitoring stations [22].

Combining GNSS measurements and tide gauge data to estimate absolute sea level changes has been discussed [23], and the research results indicated that approximately five years of GNSS observations were required to estimate vertical land motion with an accuracy of about 0.1 mm per year. This accuracy is sufficient to enhance the precision of estimating absolute sea level rises. The use of GNSS observations to measure annual sea level changes in the Red Sea has also been demonstrated, with the research showing that GNSS observations can effectively measure the impact of these sea level changes. In the northern Red Sea, the observed annual signals and predicted annual sea level loads exhibit good consistency [24]. A method for estimating ocean surface parameters using a geodetic GPS receiver was also discussed in [25]. This study indicated that the method can be effectively used to estimate sea level, wind direction, significant wave height, and peak period, offering valuable insights for oceanographic research and monitoring.

Therefore, it should be emphasized that the GNSS positioning accuracy at sea for measuring sea surface height is a crucial aspect [26].The presence of multipath effects can lead to unstable sea positioning data, necessitating the use of specialized processing techniques for using GNSS data in ocean tide measurements [27]. In particular, when using low-cost GNSS equipment, cycle slips and multipath effects are more pronounced. Consequently, developing robust methodologies for detecting outliers in GNSS positioning time series and reconstructing missing data segments constitutes a critical research imperative to enhance the reliability of tidal and wave parameter estimation [28].

In the early days, gross error detection relied mainly on the classical least squares (LS) method, which identified outliers through residual analysis. However, this method has low sensitivity to gross errors and has difficulty handling complex GNSS observation data effectively. With the development of measurement technology, gross error detection methods based on statistical hypothesis testing have gradually become mainstream [29]. Based on classical least squares theory, the method constructs test statistics that effectively reduce the error impact caused by gross errors [30]. By simulating time series of real GPS data, gross errors have been detected using both manual inspection and conventional gross error detection methods [31]. The results indicate that manual inspection is often more accurate than conventional gross error detection methods, especially in cases where the distribution of gross errors is uneven or the amount of observed data is small. However, manual inspection methods rely on experience and have difficulty transitioning to automated processing, thus limiting their application scope. In the future, with the continuous development of GNSS technology and the increasing demand for applications, gross error detection methods will continue to develop toward automation, intelligence, and multi-source fusion [32].

Currently, there are numerous methods used for identifying gross errors in GNSS coordinate time series. These include the interquartile range (IQR) criterion [33], the “3σ” criterion [34], and the median absolute deviation (MAD) criterion [35]. Compared with the 3σ and MAD criteria, the traditional IQR criterion is less susceptible to gross errors and exhibits superior robustness. However, experiments have indicated that this method may yield more false positives for data with high variability [33]. Detecting and correcting these gross errors is essential for accurately extracting tidal signals, especially under high-noise conditions. Consequently, it is imperative to identify an effective approach for eliminating periodic variation terms in GNSS time series, thereby obtaining a randomly distributed high-frequency noise sequence. This would facilitate a more accurate and efficient detection of gross errors using the IQR method.

Many approaches to processing periodic signals have been proposed, such as the singular spectrum analysis (SSA) method, the variational mode decomposition (VMD) method, and the wavelet decomposition (WD) method. SSA decomposes signals via trajectory matrix SVD, excelling in periodic component extraction and trend identification [36]. However, its performance depends heavily on the chosen window length, and it struggles with nonlinear signals and abrupt changes. The computational cost rises significantly for data processing of long time series. VMD separates signals into band-limited modes using constrained optimization, effectively avoiding mode mixing issues [37]. It requires carefully preset parameters (K, α) and has relatively high computational demands. Its performance drops noticeably when processing highly non-stationary signals with complex frequency components. WD provides excellent time-frequency analysis using various wavelet bases, making it ideal for transient detection and localized feature extraction. The results vary considerably depending on the selected basis, and spectral leakage frequently occurs for signals with overlapping frequency bands. Its computational efficiency makes it particularly suitable for real-time applications and online monitoring systems [38].

Empirical mode decomposition (EMD), introduced by Huang in 1998 [39], is a widely utilized signal processing technique that is employed in fields such as signal processing, vibration analysis, and image processing. To tackle the challenges associated with EMD, scholars have proposed subsequent methods such as ensemble empirical mode decomposition (EEMD) [40], complete ensemble empirical mode decomposition (CEEMD) [41], and complete ensemble empirical mode decomposition with adaptive noise (CEEMDAN) [42]. These methods are extensively applied in signal processing and have also garnered attention in the study of GNSS time series [43,44].

This study proposes an improved IQR algorithm for gross error detection in coordinate time series of GNSS buoy positions based on CEEMD, termed the CEEMD-IQR algorithm. By employing CEEMD to reconstruct the GNSS coordinate time series, the residual vector of the GNSS coordinate time series is obtained, and gross error discriminant statistics are constructed for the purpose of gross error detection. Reference values are derived from tide gauge observation data from synchronously deployed tide gauges. A comparison of the coordinate time series calculated using the CEEMD-IQR algorithm and the tide gauge results indicated that the newly constructed CEEMD-IQR algorithm can more effectively detect gross errors in GNSS coordinate time series. This research method offers a novel approach and methodology for tidal signal extraction in marine measurements to enhance the ability to determine the sea surface height using GNSS buoys.

## 2. Theory and Method

### 2.1. Determining Sea Surface Height Using GNSS RTK Technology

**Figure 1:**
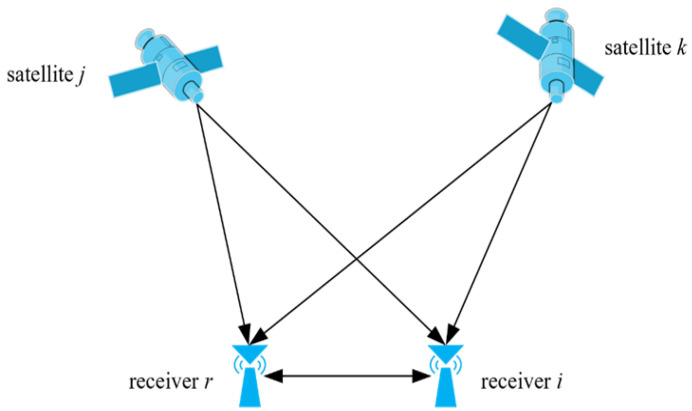
The double-difference measurement between stations and satellites for RTK technology.

Real-time kinematic (RTK) positioning is an advanced satellite positioning technology based on carrier phase observations that is widely employed for precise measurements and navigation applications within the Global Navigation Satellite System (GNSS). Utilizing the double-difference algorithm between base and rover stations, as shown in Figure 1, RTK achieves real-time positioning accuracy down to the centimeter level.

Within the RTK positioning system, a reference station and a monitoring station operate in harmony, synchronously capturing satellite signals. Assuming that the rover receiver i and the reference receiver r simultaneously track satellites j and k, the double-difference carrier phase measurement at time *t* can be expressed as

(1) $$\nabla \Delta \varphi_{ir}^{jk}(t)=\Delta \varphi_{ir}^{j}(t)-\Delta \varphi_{ir}^{k}(t)=\lambda^{-1}\nabla \Delta r_{ir}^{jk}(t)-\nabla \Delta N_{ir}^{jk}(t)+\nabla \Delta \varepsilon_{ir}^{jk}(t)$$

where ∇Δ denotes the double-deference operation; $\nabla \Delta \varphi_{ir}^{jk}$ represents the double-difference carrier phase measurement value between receiver i and r for satellite j with respect to k; λ is the wavelength of the GNSS carrier phase; $\nabla \Delta r_{ir}^{jk}(t)$ signifies the geometric distance, where the coordinate components in the east, north, and elevation directions at time t are dE(t), dN(t), and dH(t), respectively; $\nabla \Delta N_{ir}^{jk}$ refers to the ambiguity resolution; and $\nabla \Delta \varepsilon_{ir}^{jk}$ is the observation noise and error term.

In sea surface height measurement using GNSS buoys, the reference receiver is typically mounted on a fixed platform, where its elevation is known as $H_{ref}$, and the rover receiver is configured on buoys, where the height of the GNSS antenna phase center from the sea surface is calibrated as $h_{ant}$. The height of the sea surface at time t is represented as

(2) $$y(t)=H_{ref}+dH(t)-h_{ant}$$

where y(t) denotes the sea surface height at time t**,** which is calculated using GNSS technology.

The GNSS buoy continuously observes the GNSS satellite signals to estimate the height variation of the receiver antenna dH(t) with the sea level. The GNSS buoy is located in the constantly moving environment, which is complex and causes variations in GNSS observations. Gross errors are inevitably present in the dynamic positioning results. The gross errors for the positioning parameters in Equation (1) directly affect the accuracy of the sea surface height estimation for y(t). In particular, the multipath effect errors are more significant for GNSS observations at sea than on land. Multipath effects at sea cause GNSS signals to reflect and refract during propagation, resulting in additional phase delays and amplitude variations, which compromise the precision of the positioning parameters. Therefore, detecting and eliminating gross errors in positioning series is crucial for sea surface measurements using GNSS buoys.

### 2.2. IQR Gross Error Detection Method

The IQR method is a relatively robust approach for detecting gross errors. The principle of IQR gross error detection involves first arranging the data y(t) in ascending order, and then calculating three quartiles. The 25th percentile is the first quartile ($Q_{1}$), the 50th percentile is the second quartile ($Q_{2}$), and the 75th percentile is the third quartile ($Q_{3}$). The difference between the third quartile and the first quartile is referred to as the interquartile range (IQR). The IQR can be expressed as follows:

(3) $$IQR=Q_{3}-Q_{1}$$

where $Q_{1}$ represents the first quartile; $Q_{3}$ denotes the third quartile; and $Q_{2}$ refers to the second quartile. When data fall within the range $\left(Q_{1}-k\times IQR,Q_{3}+k\times IQR\right)$, the dispersion of the data is considered minimal, and these values are regarded as normal. The experimental value of k is typically set to 1.5.

### 2.3. CEEMD Method

The GNSS time series y(t) not only encompass systematic noise such as positioning errors, but also exhibit significant periodic oceanic dynamic signals, including tidal and seasonal variations. Direct application of the interquartile range (IQR) method for gross error detection in raw GNSS time series without eliminating non-Gaussian noise or temporally correlated components may not be methodologically sound. Consequently, it is imperative to implement a robust preprocessing method that effectively mitigates these systematic bias components prior to subsequent gross error detection analyses.

The EMD algorithm adaptively decomposes a signal into a series of intrinsic mode functions (IMFs) and a residual component, based on the characteristics of the signal itself. This decomposition relies on the distribution of the signal’s extrema. However, interruptions in the signal, such as discontinuities and impulse noise, can affect the selection of these extrema, leading to erroneous envelopes and resulting in mode mixing in the IMF components, thus causing them to lose specific physical meaning.

The EEMD algorithm is an enhanced version of the EMD method, designed to more robustly decompose nonlinear and non-stationary signals, thereby reducing issues such as the non-uniqueness of IMF numbers and mode mixing. The principle involves artificially adding white noise to the original signal. To counteract the effects of this added white noise, the ensemble averaging process must be increased, which inherently increases the computational cost and may even introduce errors. Hence, the CEEMD has become the preferred method for GNSS coordinate sequence processing due to its adaptive decomposition capability and mode aliasing suppression, and because there is no need for preset parameters. This can be described as shown below.

(1) A pair of opposite white noise signals are added into the raw signal $s_{0}(t)$ to derive the new signals $s_{i}^{+}(t)$ and $s_{i}^{-}(t)$:
  (4) $$\left[\begin{array}{l} s_{i}^{+}(t)\\ s_{i}^{-}(t) \end{array}\right]=\left[\begin{array}{cc} 1 & 1\\ 1 & -1 \end{array}\right]\left[\begin{array}{l} s_{0}(t)\\ w_{i}(t) \end{array}\right],i=1,2,\cdots ,m$$
(2) The signals $s_{i}^{+}(t)$ and $s_{i}^{-}(t)$ are decomposed into n IMF components, respectively, using the EMD method:
  (5) $$s_{i}^{+}\left(t\right)=\sum_{j=1}^{n}imf_{j}^{i+}\left(t\right)+r_{n}^{+}\left(t\right)$$
  (6) $$s_{i}^{-}\left(t\right)=\sum_{j=1}^{n}imf_{j}^{i-}\left(t\right)+r_{n}^{-}\left(t\right)$$
(3) The mean of the IMF components is computed with m groups of noisy signals to achieve the final CEEMD result:
  (7) $$imf_{j}=\frac{1}{2m}\sum_{i=1}^{m}\left(imf_{j}^{i+}+imf_{j}^{i-}\right)$$

### 2.4. Enhanced IQR Gross Error Detection Algorithm Based on the CEEMD Method

The GNSS coordinate time series comprises signals with various frequency components. The trend and periodic components are found predominantly in the low-frequency signals, while noise and gross errors are concentrated primarily in the high-frequency signals [45]. When employing the traditional IQR gross error detection method for preprocessing coordinate time series, the method initially models the trend and periodic components based on a harmonic function model, where the least squares method is used to obtain the residual time series data. However, the least squares method is not robust, and the residual time series obtained during modeling is susceptible to gross errors, leading to “unreliable” data when the IQR method is applied to the residual series. In this study, the IQR method was improved by including enhanced CEEMD to effectively detect the gross errors in the position series of GNSS buoys. The core methodology is as follows: CEEMD is used to decompose the raw coordinate time series and extract the low-frequency part of signals, which is reconstructed into periodic and trend components; the high-frequency signals of the raw coordinate time series are extracted as the residual time series; and the IQR method is applied to the residual time series for gross error detection, consequently identifying and eliminating the position errors of the GNSS buoys. The operational sequence is executed as described below.

**Figure 2:**
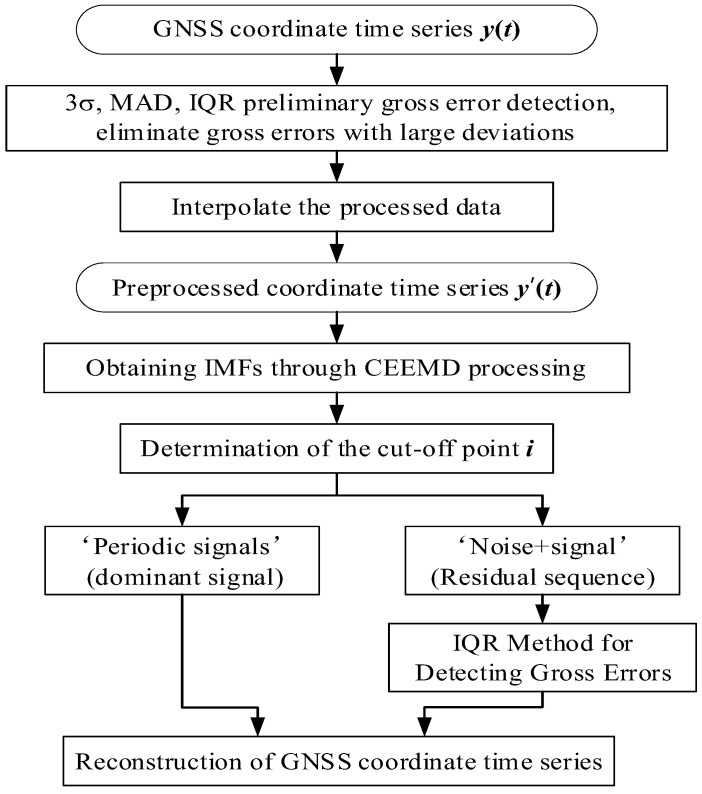
The flowchart of the enhanced CEEMD-IQR method for denoising noisy signal.

(1) The 3σ criterion method is utilized to eliminate large deviations in the raw coordinate time series y(t), preventing interference with CEEMD and reconstruction. For the outage values after eliminating gross errors, the mean interpolation method is employed to obtain a temporally continuous and complete coordinate sequence $y^{\prime }(t)$:
  (8) $$y^{\prime }(t)=y(t-1)+\frac{y(t+1)-y(t-1)}{2}$$
(2) The CEEMD method is used to decompose $y^{\prime }(t)$ and extract the high-frequency noise signals as the residual time series. For the CEEMD and reconstruction process, an appropriate method should be adopted to select the IMF components. Based on previous research, the correlation coefficient method was utilized for signal–noise separation [46]. The correlation coefficient between the IMF components and the coordinate time series $y^{\prime }(t)$ can be expressed as follows:
  (9) $$R(y^{\prime }(t),imf_{j})=\frac{\sum_{t=1}^{N}\left[y^{\prime }(t)-\bar{y}][imf_{j}-\bar{imf_{j}}\right]}{\sqrt{\sum_{t=1}^{N}{\left[y^{\prime }(t)-\bar{y}\right]}^{2}}\sqrt{\sum_{t=1}^{N}{\left[imf_{j}-\bar{imf_{j}}\right]}^{2}}}$$
  where $imf_{j}$ is the *j*th IMF component; N represents the length of the coordinate time series data; and $\bar{y}$ and $\bar{imf_{j}}$ denote the mean values of $y^{\prime }(t)$ and $imf_{j}$. The IMF component $imf_{k}$, corresponding to the first local minimum among the m correlation coefficients, is considered the boundary between noise and signal.
(3) The reconstruction with IMF components involves reconstructing the periodic signal S(t) of the coordinate time series $y^{\prime }(t)$ with the components from $imf_{k+1}$ to $imf_{m}$. After subtracting the periodic component S(t) and the trend component T(t) in $y^{\prime }(t)$, the residual time series *R*(*t*) is obtained:
  (10) $$R(t)=y^{\prime }(t)-S(t)-T(t)$$
(4) Gross errors are detected in the residual time series using the IQR method. According to the IQR criterion, when the observed value is less than $Q_{1}-1.5\times IQR$ or greater than $Q_{3}+1.5\times IQR$, it is considered a gross error and must be removed. The approach using steps (1) to (4) was named the CEEMD-IQR method and identifies all the gross errors in GNSS coordinate time series. The detailed data processing procedure is illustrated in Figure 2.

## 3. Experiment and Results

To verify the effectiveness of the CEEMD-IQR method, we generated simulated data containing trend, period, and noise components, and conducted experimental analyses using actual observation data from GNSS buoys. By comparing the results of the processing method on both simulated and measured data, the performance of the proposed method was comprehensively evaluated across different data environments.

### 3.1. Validation with Simulated Data

To further evaluate the performance of the CEEMD-IQR method in processing complex ocean altimetry data, the simulated data were initially used to conduct experimental verification. The simulated data encompassed various signal characteristics commonly observed in practical observations, such as trend terms, periodic variations, and noise interference. Subsequently, a detailed introduction to the process of generating simulated data is presented and its actual effectiveness in the application of the CEEMD-IQR algorithm was demonstrated.

#### 3.1.1. Generation of Simulation Data

A simulated time series with trend, periodic, and noise components was constructed based on the characteristics of GNSS time series with the following functional expression:

(11) $$y(t_{i})=b+v_{0}t_{i}+\sum_{m=1}^{m_{0}}[a_{m}\sin(2\pi f_{m}t_{i})+b_{m}\cos(2\pi f_{m}t_{i})]+r_{t_{i}}$$

where i represents the coordinate epoch; $y(t_{i})$ is the coordinate value of the GNSS station at time $t_{i}$; b denotes the initial position of the time series; $v_{0}$ refers to the linear velocity of the station’s movement; $m_{0}$ signifies the number of periodic signals; $a_{m}$ and $b_{m}$ represent the amplitude and frequency, respectively, of the periodic component with frequency $f_{m}$; and $r_{t_{i}}$ stands for the random noise signal.

The simulated elevation coordinate time series obtained from Equation (11) is regarded as the raw data, with the parameters set as shown in Table 1.

**Figure 3:**
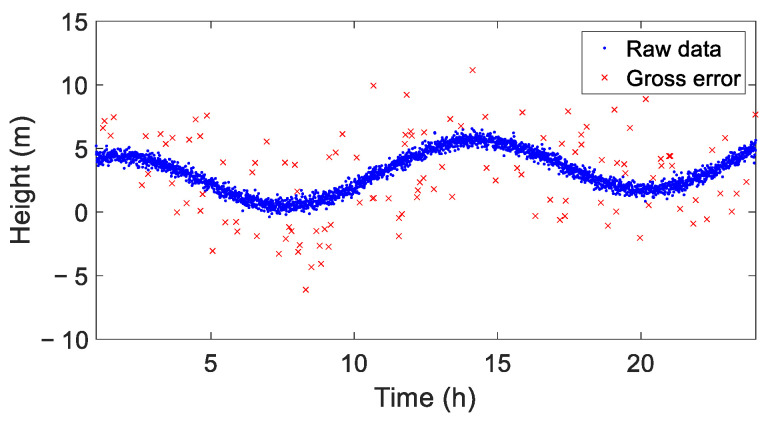
Simulated coordinate time series.

Gross errors were added to the original data. Firstly, a random error sequence following a normal distribution with a standard deviation of 6$\sigma_{wn}$ ($\sigma_{wn}$ being the noise standard deviation and $\sigma_{wn}$ = 0.4) was simulated. Then, data points greater than 3$\sigma_{wn}$ were extracted as gross errors and randomly inserted into the raw data, resulting in a GNSS coordinate time series contaminated with gross errors. The simulated time series spanned 24 h, with an epoch interval of 30 s, totaling 2880 epochs. The total number of gross errors was 125, accounting for 4.34% of the total epochs in Figure 3.

#### 3.1.2. Analysis of Simulated Data

Four methodologies, namely LS-3σ, LS-MAD, LS-IQR, and CEEMD-IQR, were employed to process the simulation data for the comparative analysis. The first three approaches are grounded in Equation (11), utilizing the least squares method to derive the residual sequence, which is subsequently used to construct gross error discriminants for error detection. In contrast, the CEEMD-IQR method employs CEEMD to eliminate trend and periodic components, thereby obtaining the residual sequence, which is then used to formulate gross error discriminants for the detection of gross errors.

**Figure 4:**
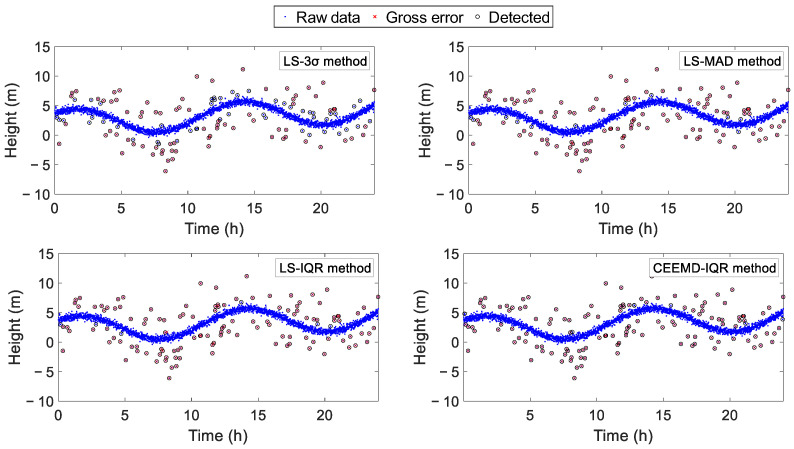
Gross error detection results using the LS-3σ method, LS-MAD method, LS-IQR method, and CEEMD-IQR method.

Figure 4 demonstrates that, although the LS-3σ method successfully identified most of the gross errors, its effectiveness diminished when detecting deviations with smaller magnitudes. In comparison, the LS-MAD, LS-IQR, and CEEMD-IQR methodologies exhibited robust detection capabilities for the vast majority of the gross errors. As quantified in Table 2, the residual analysis using CEEMD demonstrated superior detection efficacy compared with residuals derived from least squares estimation. Notably, the CEEMD-IQR method achieved an exceptional gross error detection rate of 97.6%, outperforming its counterparts. The LS-MAD and LS-IQR methods attained comparable detection rates to those of the CEEMD-IQR method, a phenomenon attributable to the simulation’s exclusive use of white noise without incorporating complex signal components such as seasonal variations or step changes.

It should be emphasized that when the a priori model is sufficiently accurate, the least squares method can precisely obtain the residuals of the simulated data, resulting in a better gross error detection performance. The number of misjudged gross errors when using the CEEMD-IQR method was significantly higher than that of the others. This phenomenon arises from two interrelated factors: (1) Identical standard deviations between the intentionally injected gross errors and background noise in the simulated time series create spectral overlap, making a subset of the gross errors that are statistically indistinguishable from noise components. (2) The CEEMD process inherently mixes high-frequency noise with gross error signatures during the generation of intrinsic mode functions (IMFs), resulting in contaminated residual sequences that simultaneously contain both error types. Consequently, the CEEMD-IQR method demonstrated increased false positive rates compared with the least squares technique since its discriminants must operate on composite signals rather than pure error residuals.

**Figure 5:**
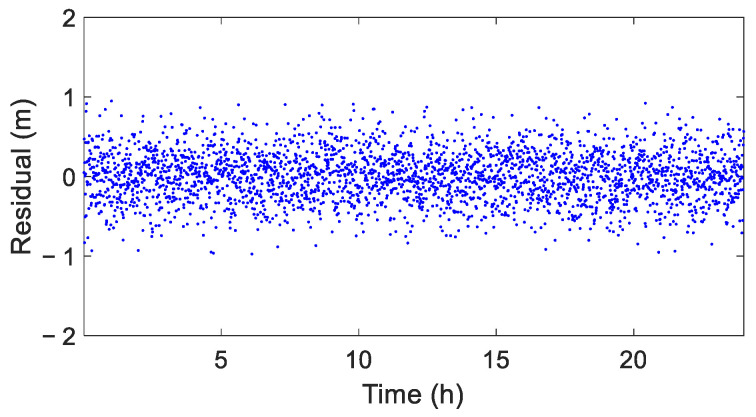
Residual sequence after eliminating gross errors using the CEEMD-IQR method.

After eliminating gross errors using the CEEMD-IQR method, the residuals obtained through CEEMD, excluding trend and periodic components, are shown in Figure 5. It is evident that the residual sequence was a white noise sequence without obvious outliers, indicating that the CEEMD-IQR method can effectively detect gross errors in the simulated time series.

**Figure 6:**
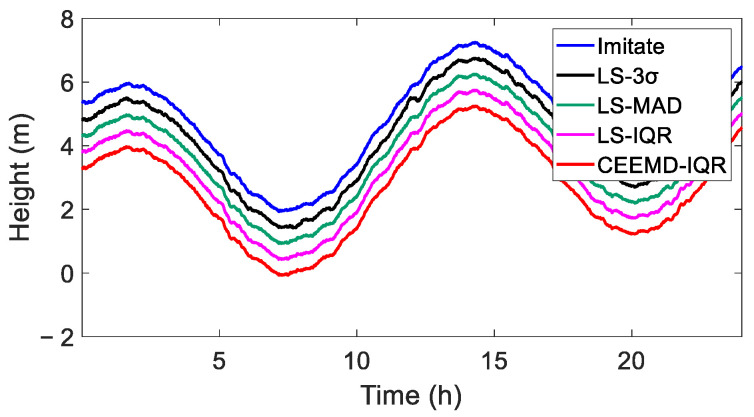
True value and elevation sequence of simulated data after removing gross errors (the curves are offset by 0.5 m to facilitate visualization and comparisons).

A comparative analysis of the LS-3σ, LS-MAD, LS-IQR, and CEEMD-IQR processing methods against the simulated truth values indicated that the four methods exhibited good consistency and are all capable of achieving satisfactory time series data, as shown in Figure 6.

To illustrate the efficacy of processing the simulated data, the coordinate time series curves were systematically displaced by 0.5 m. Figure 6 reveals that the outcomes refined using the four methods were consistent with the true value of the simulated data, exhibiting no conspicuous discrepancies. In Table 3, for the CEEMD-IQR method, the RMSE value was smaller than that of the others and the correlation coefficient was the largest. The results indicate that the CEEMD-IQR method demonstrated favorable applicability.

### 3.2. Analysis of Measured Data from GNSS Buoys

#### 3.2.1. Acquisition of Measured Data

GNSS marine buoys play a pivotal role in high-precision marine tide level measurement systems. Leveraging the data obtained from GNSS buoys enables the accurate determination of marine parameters, including tide levels and wave characteristics, as well as the on-orbit absolute calibration of satellite altimeters.

**Figure 7:**
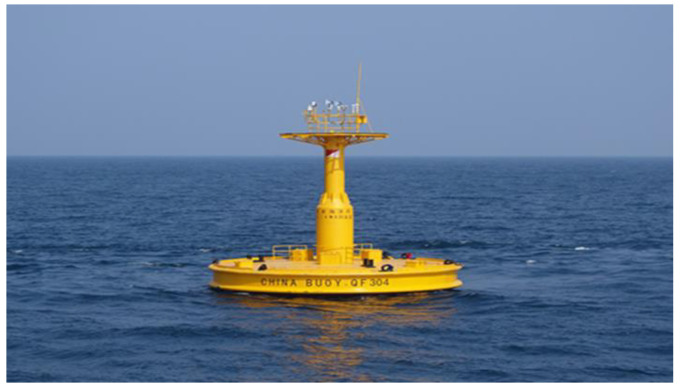
Multi-antenna GNSS buoy sea level measurement platform.

The empirical data utilized in this research were acquired in April 2023, procured in the coastal waters adjacent to Qingdao, China, by employing a GNSS buoy (Figure 7) positioned at a baseline of roughly 3 km. To ascertain the efficacy of the proposed methodology for the derivation of sea surface elevations, contemporaneous tide gauge data obtained in close proximity to the buoy were employed as a benchmark. The GNSS antenna was placed on the top of the buoy, where it was approximately 10 m above the sea surface. The position of GNSS buoys is affected by ocean waves, which present as high-frequency noise. The GNSS observation interval was set to 1 s, which allowed for high-frequency tide level measurements. A pressure gauge was adopted for measuring the real-time underwater depth of the buoys to correct for the difference in height of the GNSS antenna and the sea surface.

**Figure 8:**
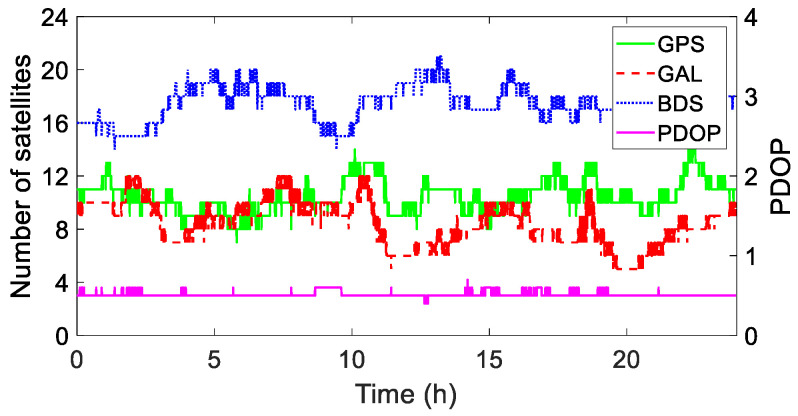
Number of available satellites and PDOP (GAL and BDS are the abbreviations for the Galileo and Beidou systems, respectively).

GPS, Galileo (GAL), and BDS satellite data were processed, and the cutoff elevation angle was set to 15° for observations. The instantaneous mode was used for fixing the ambiguity resolution with a ratio value of 3.0. The number of available satellites and the position dilution of precision (PDOP) values during the experiment are shown in Figure 8.

**Figure 9:**
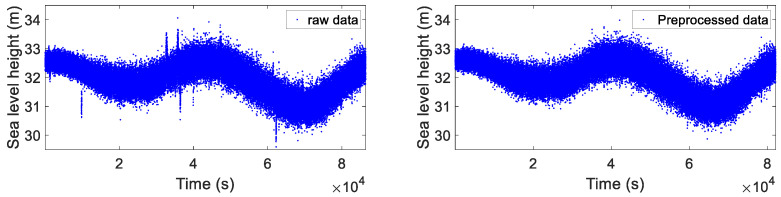
Raw data and data with unresolved ambiguities removed.

RTK technology was used to derive the GNSS coordinate time series in an upwards direction; the ambiguity fixing rate was 95.6%, indicating that the positioning accuracy was sufficient for the measurement of sea surface elevation. As shown in the raw data, significant gross errors were present due to the float ambiguity resolution, the high multipath effect, and numerous cycle slips in the sea observation data, which severely affected the precision of the results. Firstly, the coordinates with float ambiguity resolution were removed from the raw sequence to ensure a better initial time series. The total number of observation epochs decreased from 85,959 to 82,111, as shown in Figure 9.

#### 3.2.2. Detection of Gross Errors in GNSS Buoy Data

**Figure 10:**
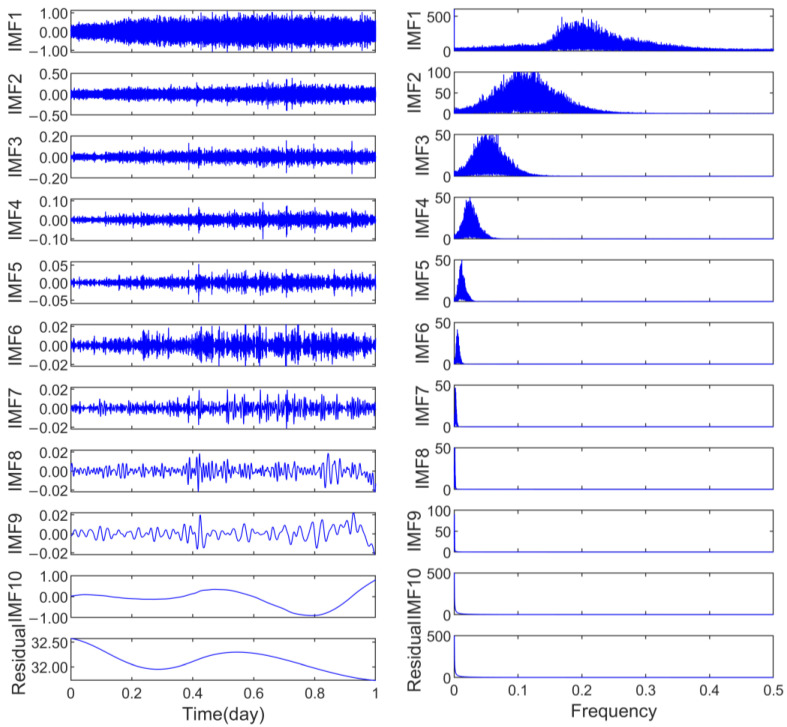
CEEMD and spectrum diagram.

The preprocessed data were subjected to decomposition using CEEMD to extract the IMF components. Subsequently, fast Fourier transform (FFT) was applied to conduct spectral analysis on the IMFs derived from CEEMD. The outcomes of this analysis are presented in Figure 10.

To identify the position of the mode-mixing components, the correlation coefficients between each IMF component and the preprocessed coordinate time series were calculated.

**Figure 11:**
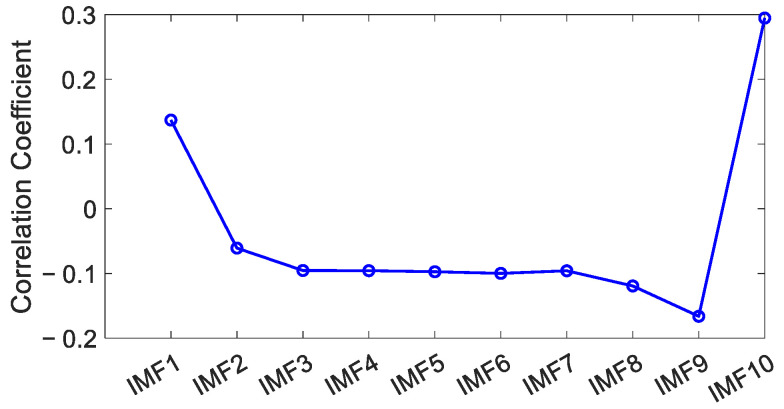
The correlation coefficients of every IMF component.

As illustrated in Figure 11, the first local minimum of the correlation coefficient was distinctly observed at IMF6 [42], which served as the demarcation point between the noise-dominated and signal-dominated components identified through the correlation coefficient method, characterizing the dynamic equilibrium state between high-frequency noise energy and low-frequency signal energy. By reconstructing the periodic signal by combining components from IMF7 to IMF10 using the CEEMD method, the residual sequence was obtained by eliminating these periodic signals and the trend components from the raw data.

**Figure 12:**
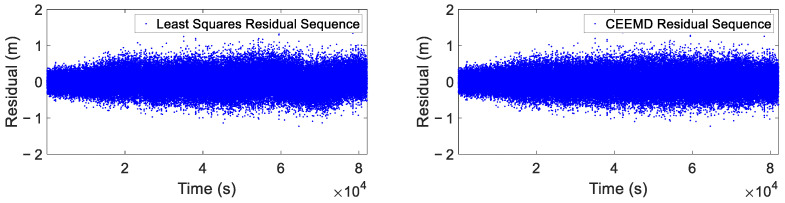
Residual sequences using least squares method and CEEMD method.

The residual sequences obtained after using the least squares and CEEMD methods to eliminate trend and periodic components in the raw data are presented in Figure 12. The traditional least squares method may have systematic deviations between its fitted curve and the true signal when obtaining residuals, which can directly affect the accuracy of residual extraction. In contrast, the CEEMD method extracts residual components through an adaptive decomposition process, which can better preserve the original features of the signal and avoid the impact of fitting biases. The results demonstrated that the CEEMD-derived residual sequence exhibited characteristics closely approximating white noise, whereas the least squares approach yielded residuals containing discernible periodic signals. This comparative analysis revealed that, although the least squares method showed susceptibility to gross errors and colored noise interference, the CEEMD method demonstrated superior capability in accurately extracting residual sequences through the effective elimination of both trend and periodic components, without requiring prior information about the signal characteristics.

The number of gross errors detected in the obtained residual sequences are presented in Table 4. The comparative analysis revealed that the CEEMD-IQR method demonstrated superior performance in gross error detection, identifying a minimum of 199 additional gross errors compared with the LS-3σ, LS-MAD, and LS-IQR methods, representing a significant enhancement in the gross error identification rate of at least 35.3%.

**Figure 13:**
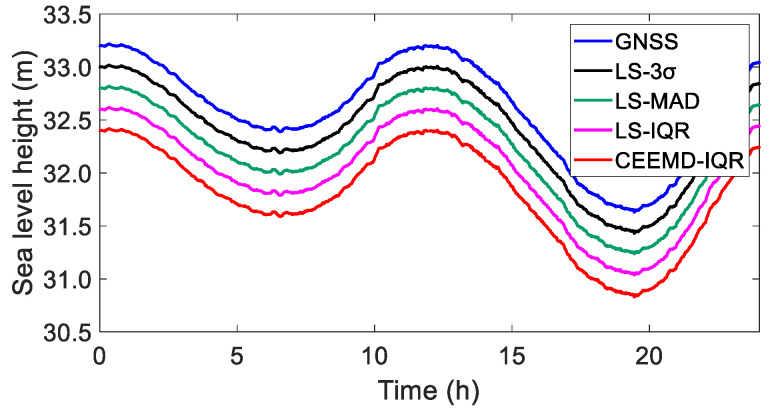
GNSS preprocessed data and reconstructed elevation sequence for the different methods (the curves are offset by 0.2 m to facilitate visualization and comparisons).

For a comprehensive evaluation, gross error detection was systematically conducted on the preprocessed dataset using four distinct methods: LS-3σ, LS-MAD, LS-IQR, and CEEMD-IQR. Following the removal of the detected gross errors, the residual sequences were reconstructed by incorporating the corresponding periodic and trend components. To validate the reconstruction accuracy, the coordinate time series generated by each method were rigorously compared with the preprocessed GNSS data. The comparative results of the coordinate time series deviations are presented in Figure 13, illustrating the performance differences among the methods.

As is evident from Figure 13, the decomposition and reconstruction process significantly enhanced the smoothness of the curves, demonstrating the effective filtration of swell-induced disturbances and measurement noise in the tidal observations. The comparative analysis revealed that the GNSS coordinate time series reconstructed using the four distinct methods maintained remarkable consistency with the preprocessed GNSS data, with no statistically significant deviations observed among the different approaches.

### 3.3. Analysis of the Sea Surface Height Accuracy Using GNSS Buoys

**Figure 14:**
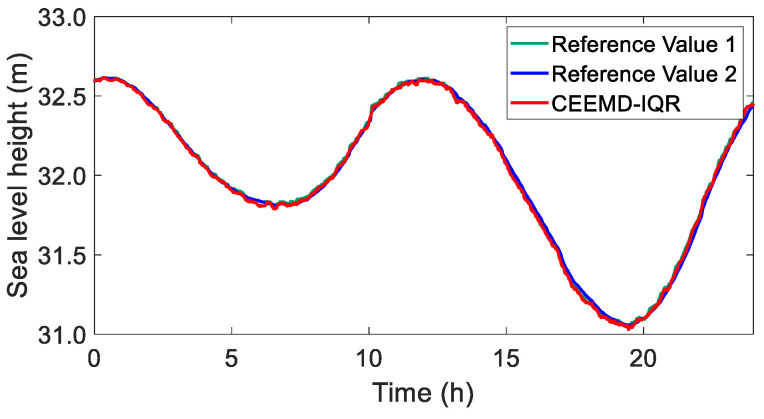
Reconstructed GNSS coordinate time series using CEEMD-IQR, GNSS preprocessed coordinate time series using CEEMD, and tide gauge coordinate time series.

To further analyze and evaluate the accuracy of sea surface height determination using GNSS buoys, the reconstructed GNSS coordinate time series obtained using CEEMD-IQR was compared with reference value 1, which was directly reconstructed using the periodic and trend components extracted using the CEEMD method, and reference value 2, which was obtained from the tide gauge. The comparative results are presented in Figure 14.

The comparative analysis demonstrated that the GNSS height series reconstructed using the CEEMD-IQR method exhibited remarkable alignment with the reference height series, maintaining consistent trend characteristics without significant deviations.

To quantitatively assess the reconstruction performance, a comprehensive evaluation was conducted by calculating the RMSE values and correlation coefficients for both the original and processed time series. These statistical metrics served as the quantitative evaluation criteria, with the detailed comparative results presented in Table 5.

The comparative analysis presented in the table revealed significant differences in performance among the methods. When evaluating against reference value 1 (the combined periodic and trend components derived from CEEMD), the CEEMD-IQR method demonstrated superior accuracy, exhibiting an RMSE value that was at least 87.9% lower than those of the other three methods. This substantial improvement can be attributed to the inherent limitations of the LS method in handling GNSS coordinate time series, where the presence of gross errors compromises the fitting accuracy, resulting in elevated RMSE values when compared with CEEMD-derived references.

When validated against reference value 2 (tide gauge measurements), the CEEMD-IQR method maintained its performance advantage, showing an RMSE reduction of at least 13.2% compared with the alternative methods. This consistent performance across different reference datasets confirmed the robustness of the CEEMD-IQR approach in gross error detection.

The correlation analysis found that all four methods demonstrated a strong correlation with both reference datasets, indicating their fundamental capability to extract accurate tidal elevation information. However, the CEEMD-IQR method consistently achieved the highest correlation coefficients, suggesting that its reconstructed tidal information exhibited the closest agreement with real-world observations. This superior performance underscores the method’s effectiveness in preserving the integrity of tidal signals during the reconstruction process.

It is also important to highlight that, in this experiment, the total number of data epochs reached 82,111, with an ambiguity fixing rate of 95.6% during data processing. Among the accuracy evaluation metrics, the RMSE indicator is not particularly sensitive to a small number of gross errors. As a result, the performance difference observed in the RMSE2 results was only at the millimeter level. Nevertheless, this improvement demonstrates that the enhancement in gross error detection achieved a minimum increase of 35.3%.

## 4. Conclusions

GNSS buoys are effective high-precision ocean observation sensors that can measure the absolute height of the sea surface. Its accuracy in determining the sea surface height depends on the GNSS positioning performance and reliability. Consequently, developing robust methodologies for the timely detection of gross errors in GNSS positioning outputs has become imperative to ensure the reliability of sea surface height measurements.

This study presents a comprehensive methodology for GNSS time series analysis and marine buoy monitoring that employs advanced signal processing techniques. The research methodology comprises three main components: simulation analysis, measured data processing, and comparative validation.

Based on the characteristic components of GNSS time series, we constructed a synthetic dataset incorporating trend terms, periodic terms, and noise terms, subsequently introducing controlled gross errors for method validation. Through a comparative analysis of the residual sequences obtained via both least squares (LS) and CEEMD methods, the CEEMD-IQR approach demonstrated superior performance in gross error detection. The CEEMD-IQR method achieved the highest correlation coefficient (0.9997) with the true value of simulated data while maintaining the lowest RMSE, confirming its effectiveness in gross error detection in GNSS data.

For practical application, the GNSS marine buoy data were processed to mitigate the effects of ocean swell and noise contamination. Residual sequences were extracted using both traditional LS and CEEMD methods, followed by gross error detection employing four distinct methodologies: CEEMD-IQR, LS-3σ, LS-MAD, and LS-IQR. The reconstruction process integrated the purified residuals with the corresponding periodic and trend components. The experimental results demonstrated that the CEEMD-derived residual sequences exhibited characteristics closer to white noise compared with those of the traditional LS methods, showing enhanced resistance to gross errors and colored noise interference. The CEEMD-IQR method consistently identified a greater number of gross errors across all the test scenarios.

The study focused on marine buoy monitoring, evaluating GNSS measurement effectiveness through a rigorous comparison with high-precision tide gauge data. A rigorous comparative analysis was conducted on the reconstructed data from four distinct methodologies, incorporating both statistical accuracy metrics (RMSE) and a correlation analysis of the coordinate time series. The evaluation utilized two reference standards: (1) the reconstructed values with the intrinsic periodic and trend components derived from CEEMD, and (2) the tide gauge measurement results.

The analysis revealed that the CEEMD-IQR method consistently achieved the best performance metrics, demonstrating superior reconstruction quality, as demonstrated by the highest correlation coefficient and the lowest RMSE values among all the evaluated methods. Specifically, the method attained a sea surface measurement accuracy of 1.51 cm, representing a significant advancement in measurement precision. When validated against tide gauge reference data, the CEEMD-IQR method exhibited an RMSE reduction of at least 13.2% compared with the other approaches.

These results substantiate two key advantages of the CEEMD-IQR method: (1) its robust capability in precise gross error elimination, and (2) its significant enhancement of ocean altimetry measurement accuracy using GNSS buoys. The method’s consistent performance across the different evaluation metrics and reference standards underscores its practical value in operational marine monitoring applications, particularly in scenarios requiring high-precision sea surface measurements.

## Figures and Tables

**Table 1:** Parameter values used for simulation.

T/h	b/m	$v_{0}$/(m/h)	$a_{1}$/m	$b_{1}$/m	$a_{2}$/m	$b_{2}$/m	$f_{m}$
24	1	0.1	1	1	0.6	0.6	0.08

**Table 2:** Comparison of gross error detection results for different methods.

Method	Number of Gross Errors	Gross Error Detection Rate (%)	Number of Gross Errors and Misjudgments
Ls-3σ	76	60.8	0
Ls-MAD	114	91.2	0
Ls-IQR	119	95.2	1
CEEMD-IQR	122	97.6	3

**Table 3:** Accuracy analysis of the four methods using simulated data.

Method	Ls-3σ	Ls-MAD	Ls-IQR	CEEMD-IQR
RMSE (cm)	3.89	1.78	1.68	1.64
Correlation	0.99968	0.99993	0.99994	0.99994

**Table 4:** Comparison of gross error detection results of the various methods.

Method	Ls-3σ	Ls-MAD	Ls-IQR	CEEMD-IQR
Number of gross errors	359	563	560	762

**Table 5:** The accuracy for gross error detection using the different methods.

Method	RMSE1 (cm)	RMSE2 (cm)	CORR1	CORR2
LS-3σ	4.31	1.88	0.99589	0.99912
LS-MAD	4.29	1.71	0.99593	0.99911
LS-IQR	4.29	1.72	0.99586	0.99911
CEEMD-IQR	0.52	1.51	0.99981	0.99913

Note: RMSE1 and CORR1 were calculated using reference value 1, while RMSE2 and CORR2 were calculated using reference value 2.

## Data Availability

The data presented in this study are available on request from the corresponding author.
